# A Unique Presentation of Peripheral T Cell Lymphoma: Diagnosis Behind the Deceiving Dry Tap

**DOI:** 10.7759/cureus.56120

**Published:** 2024-03-13

**Authors:** Monisha Rita Jayaraman, Shobini Vishali, Sarah Grace Priyadarshini

**Affiliations:** 1 Department of Pathology, Saveetha Medical College and Hospital, Saveetha Institute of Medical and Technical Sciences, Saveetha University, Chennai, IND

**Keywords:** non-hodgkin's lymphoma, bone marrow, peripheral t cell lymphoma, myelofibrosis, lymphoma

## Abstract

Numerous neoplastic, viral, hematological, or metabolic conditions that affect the bone marrow might result in secondary myelofibrosis. The bone marrow aspirate results in a dry tap and bone marrow biopsy reveals significant fibrosis replacing the normal hematopoietic cells. This is an intriguing example where a bone marrow aspirate showed a dry tap, and subsequent examination revealed a peripheral T cell lymphoma (PTCL). PTCLs are an aggressive group of non-Hodgkin's lymphoma. They often present as peripheral lymphadenopathy. The unique presentation of this case is explored in this article.

## Introduction

Peripheral T cell Lymphoma (PTCL) is an aggressive tumor that frequently manifests as peripheral lymphadenopathy, but it can also develop extra nodally, affecting the skin, gastrointestinal tract, lungs, or, very infrequently, the central nervous system [1]. It has also been documented that PTCL can occasionally manifest with pruritus, eosinophilia, and hemophagocytic syndrome [1,2]. Secondary myelofibrosis complicating T cell lymphoma is extremely rare, and the pathogenesis behind the development of myelofibrosis remains unknown in many of the reported cases [1-3]. There are very few reports of advanced PTCL cases that affect the bone marrow, causing fibrosis while disguising themselves as myelofibrosis. Here, one such instance of PTCL, presenting as myelofibrosis, is explained along with a brief discussion on the pathogenesis.

## Case presentation

A 52-year-old male came to the outpatient department with a fever and cough for five days and generalized body pain for three days. The fever was continuous and not associated with chills or rigor. Cough was associated with white sputum production. The patient had no significant past or family history. The patient was admitted to the hospital with a fever for evaluation. A complete blood count was done, which showed a hemoglobin (Hb) count of 7.8 g/dL, a total red blood cell (RBC) count of 3.0 million/mm^3^, a platelet count of 0.51 lakhs/mm^3^, and a total leucocyte count of 4790 cells/mm^3^.

Peripheral smears showed normocytic normochromic RBCs showing moderate anisopoikilocytosis with elliptocytes and tear drop cells with polychromatic macrocytes. White blood cell counts were normal, but a reduced number of neutrophils was noted. Platelet counts were also found to decrease. The smear was negative for malarial parasites and microfilariae. An impression of normocytic normochromic anemia with neutropenia and thrombocytopenia was given. A routine urine examination was normal. The stool examination was negative for occult blood. The erythrocyte sedimentation rate was increased to 120 mm/hr, and serum lactate dehydrogenase was also increased to 332 U/L. Tests were done for dengue Ns1 antigen, dengue IgM, leptospira IgM, and scrub typhus but turned out to be negative. Renal function tests and liver function tests were within normal limits. HbsAg, HCV, and HIV serology were negative. A bone marrow aspirate was done, which resulted in a dry tap. Computed tomography (CT) thorax plain and contrast enhanced were done, which showed multiple enlarged homogenously enhancing lymph nodes in the bilateral axilla, showing a central area of necrosis. The largest lymph node measures 2.5 x 1.6 cm in the right axilla. A few prominent mediastinal lymph nodes were also noted. Contrast-enhanced CT of the abdomen showed a mild increase in spleen size measuring 13.5 cm and multiple enlarged, homogenously enhancing retroperitoneal lymph nodes, of which the largest measured 1.9 x 1.2 cm in the para-aortic region. Heterogeneously enhancing enlarged conglomerate lymph nodes were noted in the bilateral inguinal region, with the largest measuring 3.9 x 2.6 cm on the right side.

**Figure 1:**
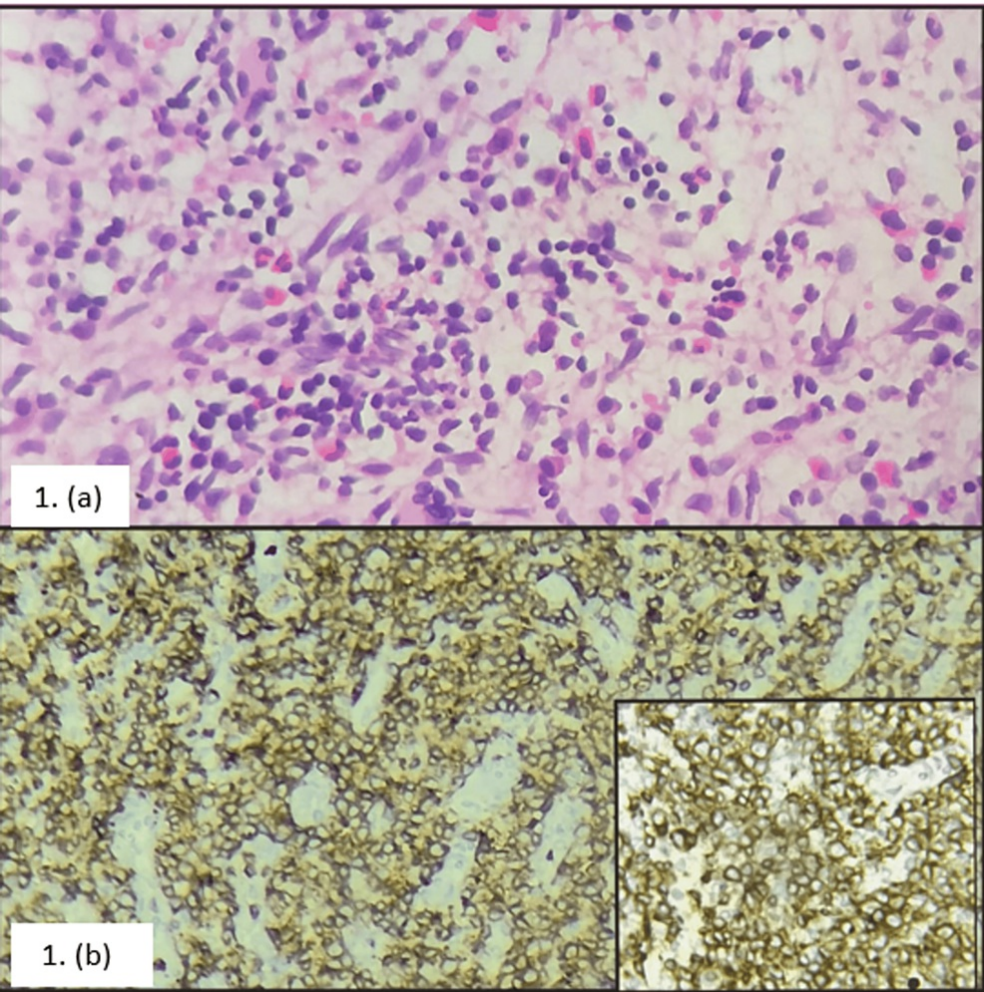
Inguinal lymph node biopsy with lymphocytes showing CD3 positivity (a) Inguinal lymph node showing diffuse infiltration of medium- and large-sized lymphocytes. (b) Lymphocytes showing CD3 positivity. Higher magnification inset.

A biopsy was taken from the inguinal node, which showed linear fragments of lymphoid tissue with diffuse infiltration of medium- and large-sized lymphocytes, which have hyperchromatic irregular nuclei with increased mitotic activity. Figure 1a shows the intervening small lymphocytes, eosinophils, proliferating blood vessels, and areas of edema and necrosis. Immunohistochemistry was done, which revealed ki67 strong positive, CD3 strong positive in the large lymphoid cells, CD20 negative in the large lymphoid cells, Tdt negative in the large and small lymphoid cells, and MUM1 strongly positive in 30% of the large lymphoid cells. Figure 1b shows the inguinal lymph node biopsy with its immunohistochemistry for CD3.

**Figure 2:**
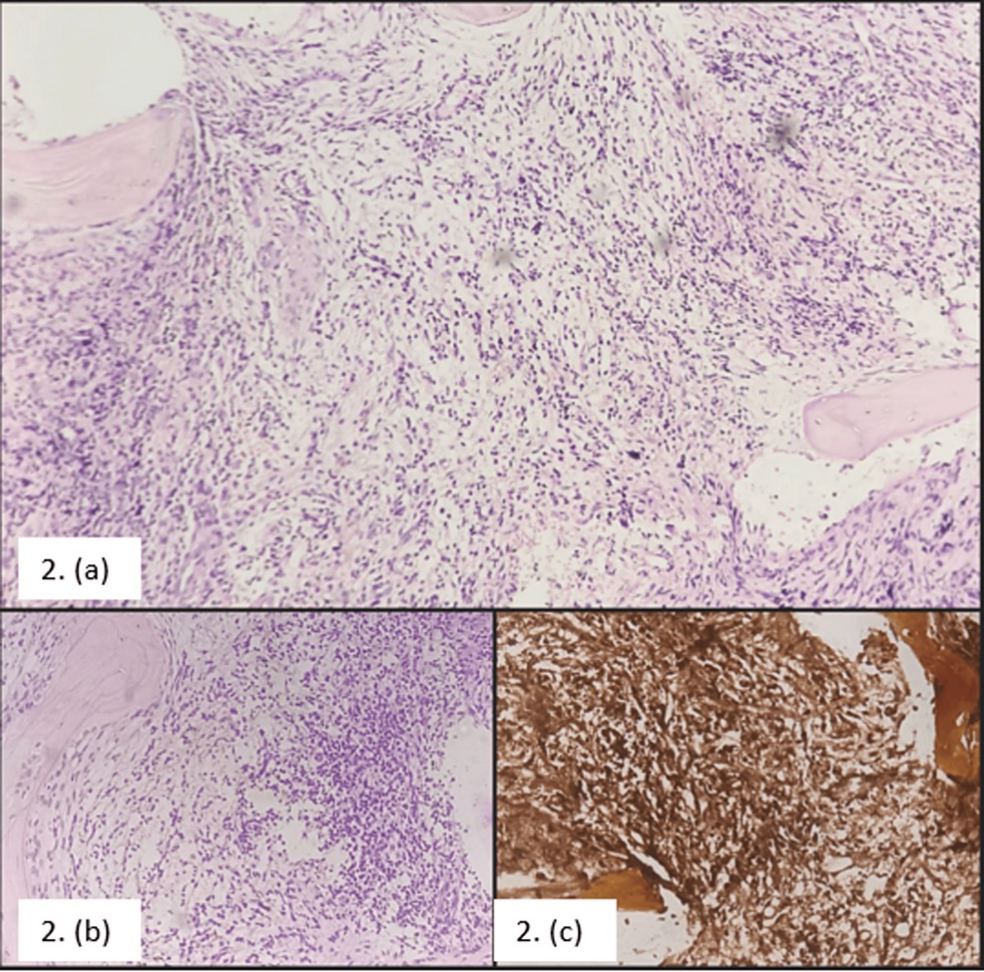
Bone marrow biopsy showing nodular infiltration of atypical cells and marrow space obliteration by fibrosis highlighted by reticulin stain (a) Bony trabeculae with cellular marrow spaces obliterated by fibrosis and infiltration of atypical small- to medium-sized cells. (b) Bony trabeculae showing nodular infiltration of small- to medium-sized cells. (c) Reticulin stain of the bone marrow showing fibrosis.

A bone marrow biopsy was taken for the patient, which showed bony trabeculae with cellular marrow spaces obliterated by dense, diffuse, coarse fibrosis and vague nodular infiltration by atypical small- to medium-sized lymphoid cells. There is reduced erythropoiesis and megakaryopoiesis. Myelopoiesis is relatively preserved. Thus, a bone marrow biopsy exhibited features of non-Hodgkin’s lymphoma infiltration with myelofibrosis. Figure 2 shows sections from the bone marrow biopsy with reticulin staining of the same which showed marrow fibrosis of grade 4.

**Figure 3:**
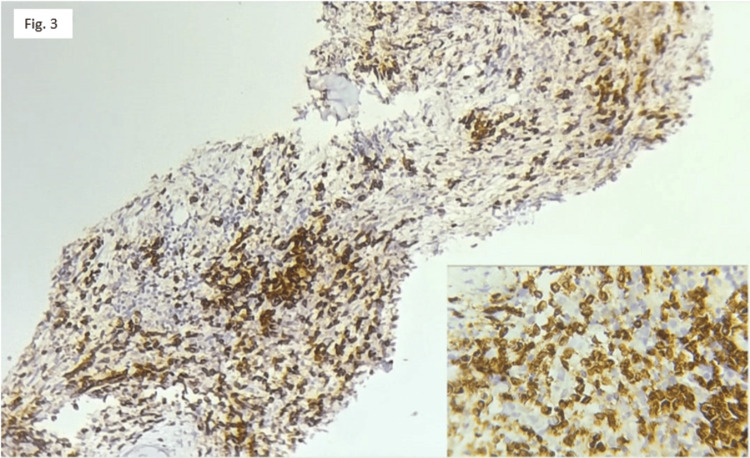
Atypical cells in the bone marrow showing CD3 positivity Bone marrow with the infiltrating cells showing CD3 positivity. Inset showing a higher magnification image.

Figure 3 shows bone marrow infiltrated by atypical cells which were positive for CD3 immunohistochemistry. 

The patient underwent eight cycles of chemotherapy, and the blood parameters have greatly improved. A recent hemogram showed a hemoglobin level (Hb) of 9.5 g/dL, a total red blood cell count of 3.34 million/mm^3^, a platelet count of 1.38 lakhs/mm^3^, and a total leucocyte count of 8750 cells/mm^3^.

## Discussion

PTCL is a disease that occurs during the sixth to seventh decade of life [1]. Various studies show that systemic symptoms like fever, night sweats, and weight loss, known as the B symptoms, are common in PTCL and occur in almost half of the patients [1]. Most patients are in stage III or IV at the time of diagnosis and present with extranodal involvement. These extranodal sites include the bone marrow, liver, spleen, and lung [2,3]. Sometimes the initial presentation can be a cytokine-related paraneoplastic syndrome, which manifests as pruritus, eosinophilia, and hemophagocytic syndrome [1,2]. Rarely do patients present with an immunological condition such as Hashimoto thyroiditis or immune thrombocytopenic purpura. A bone marrow biopsy is not essential in the diagnosis of PTCL [1,3]. There may be histological similarities and overlaps between various reactive T cell proliferations and other cancers. Moreover, PTCL typically results in secondary marrow abnormalities that could mask the neoplastic infiltration [2-4]. A thorough integration of data from the peripheral blood, bone marrow aspirates, biopsy results, and clinical characteristics is often required for the diagnosis. Examination of the histology of bone marrow biopsy in light of the updated WHO classification in PTCL is required to avoid the diagnostic pitfalls. PTCLs are aggressive malignancies with a low five-year survival rate and poor therapeutic efficacy. T cell lineage is associated with poor prognosis, and other factors that favor poor prognosis include older age (more than 60 years), high serum lactase dehydrogenase levels, and bone marrow involvement [3-5].

Primary myelofibrosis is a clonal myeloproliferative neoplasm and is characterized by the deposition of fibrous tissue in the marrow when there is no antecedent; that is, there’s no precursor malignancy [2]. Secondary myelofibrosis, on the other hand, is secondary to a disorder, as the name suggests. It usually occurs following essential thrombocythemia or polycythemia vera, or rarely, as in this case, a T cell lymphoma. The development of secondary myelofibrosis is thought to be closely related to the lymphoma invasion. However, the pathogenesis of myelofibrosis is unknown [3,4]. Several sources identify various growth factors as the promoters of fibrosis [2,3]. The growth factors discussed are transforming growth factor-β (TGF-β), basic fibroblast growth factor (b-FGF), vascular endothelial growth factor (VEGF), and tumor necrosis factor-α (TNF-α) [2,4-5]. The megakaryocytes and monocytes are the main sources of these growth factors, which in turn cause stromal proliferation, resulting in myelofibrosis [4,5]. TGF-β produces cytokines, which also contribute to fibrosis by inducing an immune and inflammatory response [1,6]. TGF-β also promotes the production and accumulation of extracellular matrix proteins such as fibronectin; types I, III, and IV collagen; and hyaluronic acid. Thus, TGF-β may be particularly important in the development of myelofibrosis in T cell lymphoma [5,7-9].

## Conclusions

In brief, the current instance of PTCL showcases a rare connection to myelofibrosis, characterized by extensive fibrosis in the bone marrow. To shed light on the development of secondary myelofibrosis in the absence of an elevated presence of megakaryocytes in the bone marrow, it is imperative to conduct further investigations and gather more case reports. Even though rare, it is essential to consider T cell lymphoma as one of the differential diagnoses while evaluating a case of myelofibrosis.
